# Dignity and Deferral Narratives as Strategies in Facilitated Technology-Based Support Groups for People with Advanced Cancer

**DOI:** 10.1155/2012/647836

**Published:** 2012-02-22

**Authors:** Annette F. Street, Kate Wakelin, Amanda Hordern, Nicola Bruce, Dell Horey

**Affiliations:** ^1^Cancer and Palliative Care Studies, La Trobe University, Bundoora, VIC 3086, Australia; ^2^Telephone and Internet Support Groups, Cancer Information and Support Service, Cancer Council Victoria, Carlton, VIC 3053, Australia; ^3^Cancer Information and Support Service, Cancer Council Victoria, Carlton, VIC 3053, Australia; ^4^Research Associate, Health Issues Centre, Bundoora, VIC 3086, Australia; ^5^Faculty of Health Sciences, La Trobe University, Bundoora, VIC 3086, Australia

## Abstract

This paper examines the value of facilitated telephone and online support groups for palliative care. Telephone interviews were conducted with twenty people living with advanced cancer who had participated in either a telephone or online support group facilitated by the Cancer Council Victoria, Melbourne, Australia. Two dominant participant narratives emerged: a focus on dying with dignity or an interest in deferring discussion of death and dying to focus on the present. Despite the different approaches, participants found the technology-based support groups to be accessible and safe environments in which to discuss difficult topics in privacy. Technology-based strategies provide opportunities for health professionals to provide social and emotional care to more people by moving beyond individualised care and facilitate peer-to-peer support at the end of life, especially to those with specific needs. Such options are feasible for palliative care services to set up and acceptable to a group of clients, especially for younger clients or those socially or geographically isolated.

## 1. Introduction

Like other high-income western countries, Australia has an ageing population and an associated increasing prevalence of cancer. In 2004, fifteen in every thousand Australians had been diagnosed with cancer in the previous five years, and, by 2010, one in two Australian males and one in three females were at risk of a cancer diagnosis before the age of 85 years. While relative survival has increased significantly, changing demographics means the number of deaths from cancer will continue to rise [5]. As in other countries [6], these changes in Australia will have important consequences for the way that health services for people at the end of life are delivered. It is unlikely to be feasible to extend current specialist palliative care to meet the anticipated demand, instead mainstream services need to acquire both skills in palliative care and understanding of the needs of people at this stage of life as part of a broader palliative approach. Recognition of the limits of existing services, both in terms of providing timely access and in meeting future palliative care needs, has prompted Australian governments to adopt and promote a health-promoting approach to palliative care designed to support Australians to live well until they die [7]. In the past, most palliative care support has been directed at individuals and their families, but as people with advanced cancer live longer in the community, more supportive care alternatives are sought.

One option is group-based support. Reported in the literature since the 1970s [8], support groups have long been considered beneficial for people experiencing cancer, as they provide opportunities to actively discuss and contemplate difficult feelings to support hope and alleviate anxiety [9]. Cancer support groups facilitated by health professionals are an important development. In eight randomised trials of peer support groups for people with cancer, only two, both involving group online support facilitated by health professionals, showed a reduction in psychological distress and improved perceptions of support and self-care [10].

Facilitated support groups are usually conducted face-to-face, and over twenty randomized studies demonstrate the efficacy of such a model in terms of therapeutic and social outcomes [11]. However, a face-to-face approach assumes that people can attend groups in person, whereas this can be a serious limitation for many palliative care clients who may be undergoing regular palliative treatment, are frail or fatigued, geographically isolated, hospitalized, or who simply prefer anonymity [1]. Technology-based options, based on online or telephone access, were adopted to address such issues [2] and appear to have considerable potential in providing social and emotional support for people in the palliative stage of their care.

Individualized telephone support is a mainstay of cancer information and support services internationally, with the advent of the mobile phone increasing the flexibility of these services. The extension of support groups onto the Internet was an obvious development to facilitate sharing experiences among cancer patients and give wider access to psychosocial support resources [10, 12]. Significant usability improvements since the basic moderated “Usenet newsgroups” of the mid-1980s have led to today's sophisticated capability of synchronous chat rooms. Comparison of online group therapy and face-to-face therapy has found an analogous psychodynamic structure in the groups and similar curative factors, although the anonymity of the online group allowed more self-disclosure and reported greater interpersonal closeness [12].

The Cancer Information and Support Service (CISS) of the Cancer Council, Victoria (in Australia), provides a comprehensive range of supportive care services for those affected by cancer. In response to service evaluations, senior staff explored the potential for technology-based solutions to address an identified need for time-limited, facilitated support groups suitable for urban, rural, and remote communities. A model for technology-based support groups was piloted in 2007, and telephone and online options have continued. People with cancer who ring the CISS Helpline looking for support are informed about telephone and online support groups and other forms of peer support. Prospective participants who express interest are phoned by a palliative care nurse facilitator to assess their suitability for involvement. Some require more intensive services such as counselling. Prior to the start of any technology-based support group, members are given detailed information including simple ways to deal with technical difficulties and tips for effective group work. Group members are encouraged to contribute a “profile” with their age, diagnosis, and participation goals, which are circulated. For advanced cancer groups (where much can change over the course of a fortnight), nurses make check-in calls to assess availability and to see if other issues have arisen. Six facilitated sessions are conducted fortnightly for each online support group over two to three months. Each session lasts from 60 to 90 minutes each and involves six to eight participants and two palliative nurse facilitators. The online support groups meet in a password protected chat room, using text input. The author of each text posting is indicated by a self-selected pseudonym. Telephone groups meet as a teleconference for the cost of a local call. Participants state their name (or pseudonym) prior to speaking. Members of both types of group are encouraged to keep a journal to record feelings and thoughts about the meetings. Following each meeting, the facilitators prepare a group-specific newsletter or online blog that recounts the issues discussed, provides follow-up information about topics raised, and reminds participants of the next meeting date. Newsletters also contain a question to aid reflection and journal writing and to prompt conversation in the following session.

In this paper, we report the research findings from a qualitative interview study that explored the experiences of adults living with advanced cancer who took part in some of these facilitated time-limited technology-supported groups. The aim was to investigate motivations for involvement in these groups, expectations of group involvement, and the type of strategies employed to provide support within the groups.

## 2. Methods

### 2.1. Study Design

This interview study used qualitative content analysis. Qualitative content analysis is one of a number of methods increasingly being used in nursing research to extend theory to answer practical rather than philosophical questions [13, 14]. As there is a body of knowledge about telephone support and chat groups in other contexts, we used directed content analysis to identify core consistencies and manifest meanings [8]. The key themes from this literature were used to inform the interview schedule and coding scheme focused on the topics of motivations, expectations, and supportive strategies.

### 2.2. Ethics

The research study was conducted with the understanding and consent of all participants and granted ethical clearance by the Human Ethics Research Committee of the Cancer Council Victoria.

### 2.3. Study Sample

After ethical approval was given in 2009, members of the next six online or telephone support groups due to be contacted for routine postgroup follow-up calls were invited to participate in interviews about their experiences. Material about the study was forwarded to all those who indicated interest. Not all members of the six groups could be contacted, and four were known to have already died. Attempts were made to contact 26 people in total—12 from telephone groups and 14 from online groups. Two did not return calls, and four people indicated they were too ill to take part. The remaining 20 (77% of those with attempted contact) agreed to take part. Research assistants experienced in providing telephone support to cancer clients conducted the interviews.

### 2.4. Data Collection

After confirming consent verbally, interviews were conducted in a conversational style by telephone. The interviews explored participants' reasons for involvement in a technology-based support group, their expectations of group support, and any supportive care strategies used. Participants related stories of their experiences within support groups, how expectations were met, their sense of group life, and specific memories of meetings that described their overall feedback. Demographic data were also collected. All interviews were audiotaped, transcribed, and stored securely.

### 2.5. Data Analysis

The analysis was guided by directed qualitative content analysis [13, 15] that allowed exploration of the interview data using the predetermined questions related to reasons for involvement, expectations of group support, and supportive care strategies [16].

Qualitative texts contain multiple meanings [17], so after clustering the emergent codes to disclose the manifest content, we conducted a second-level analysis to explore the latent meanings that had emerged from the open-ended conversational style of the interviews [8]. Accordingly, the data were initially analyzed in-depth by three researchers independently with emergent categories contrasted and compared. We examined the whole interviews along with the code clusters to explore latent themes such as (underlying narratives, meeting the needs of palliative care patients, and building a group life). These latent themes were further grouped to explore their relationships with social narratives around death and dying. The face validity of the narratives was confirmed with the facilitators of the support groups.

## 3. Results

### 3.1. Study Sample

The study sample comprised 20 people; ten who took part in an online support group and ten who took part in a telephone support group. Participants were aged between 22 and 65 years (mean = 49 years) and had a diagnosis of advanced cancer. These varied from common cancers such as breast and prostate cancer to less well known such as a rare form of sarcoma. Four participants (all female) were aged less than 40, and four participants (all male) were aged over 60.

More women (*n* = 14) than men (*n* = 6) participated in the study, consistent with the gender balance of the support groups. Participation was evenly divided between those who had been in online or telephone groups.

Six of the ten people from the telephone groups had no prior experience with teleconferencing but reported no substantial difficulties. Five of the ten participants from an online group reported experience with chat rooms and using the Internet. Of the others, one had little Internet experience and used the first session to get used to being online. Four other participants required initial technical assistance from the facilitator.

### 3.2. Reasons for Involvement

Practical needs prompted people's decisions to take part in technology-based support groups at this palliative stage of their illness. Participants joined a technology-based group because of their health status. Three participants from telephone-based groups, and four from online groups, cited being too ill as their primary reason for seeking this type of support. The nature of their illness meant many of those interviewed were undergoing palliative treatment or were in hospital when taking part in their support group. Typical comments were “*I'm not always feeling well or have the energy*” and “*I've been in and out of hospital for chemo and pain control*”. The power of using remote technology to link to a support group was captured by a participant who said in their interview “*I've been too sick in hospital. I'm in hospital now.*”

Other practical factors also drove the decision to use a technology-based group. One participant explained “*Well I live in a country town and although the next door town has a support group ours does not.*” For one woman, a single parent, attendance at face-to-face support groups was difficult to organize whereas she could find time to take part in an online support group from her home. Another was pleased that she “*did not need to get dressed-up*” to meet others.

### 3.3. Expectations of Group Support

Several participants wanted to be in a group with others at either a similar cancer stage or relevant life stage. One person said: “*In the town where I live at the moment … there is not anyone here with anything similar to what I've had.*” Other participants explained that they felt unwelcome or awkward in other cancer support groups where members were in earlier stages of the disease: “*I went back to my support group and when I told them my cancer had spread they were just horrified.*” Life stage was also important. Some participants who were young adults wanted to talk about their work identity, about leaving young children, about “unfinished business” in terms of unfulfilled life plans.

Participants felt comfortable and could talk freely about their emotions without the possibility of upsetting someone they loved. The freedom to express feelings reduced their sense of isolation, and sharing problems diminished them. One participant related how the isolation he experienced meant his problems became larger; when he heard about other people's problems and how they could be surmounted, he was able to see his situation in a new context:

*“to have other people that understand what you've been through, not because they've read about it or studied or they're experienced in treating people with it, but people that have lived through the crappy part of it. It was just invaluable.”*

An important goal for some participants was not to be identified, either because of personal circumstances or because of their public profile. It was common for participants to relate how they previously felt inhibited in face-to-face support groups and felt unable to vent their full range of emotions in those environments. The absence of visual cues did not affect interactions between group members; on the contrary, without visual cues some participants reported that as it made it possible for them to express deeply personal and emotional issues. One participant, who had previously attended a face-to-face group and found it unhelpful, said that “*being unseen*” was the main advantage of the online support group. Another participant who lived in a small town was concerned that if it was widely known that he was suffering from advanced prostate cancer, he and his family may be further isolated by the local community. Parents of young children expressed appreciation of silent online conversations that enabled them to express sentiments that would distress their children if overheard.

Participants of the online groups talked of learning to connect the disparate lines that appear on the screen to form a conversation thread and appreciated the use of emoticons to quickly depict mood. Some participants who were involved in online support groups explained how their physical body would respond to the emotion within these threads, describing moments when they would type their account whilst crying “*being totally engaged.*” The online discussions enabled participants to live in the moment and receive support at that time:

“*One time, the topic they were all talking about I found really upsetting and so I just stopped typing* ‘*cause I was crying so much. It was great because someone asked “are you OK?” … Then afterwards I'd cry and cry, but then I'd feel so much better about myself the next day.” *

Reading other stories on screen was seen as an additional benefit as they could be reread. One woman appreciated reading the details of another's experience of managing the side effects of chemotherapy. As she read the other woman's words, she recognised her own experiences and suddenly realized how she could be more in control of unpleasant side effects after her next hospital visit. What was not anticipated by participants was the closeness that would build between group members, *“It was the experience of the group with a common peril which I really enjoyed.*” Within the support group, they were not different, or at any particular disadvantage compared with the others and so were not defined by their illness.

Most of those interviewed expressed some hope or desire to meet with others from their support group or at least to hear about their progress; a few suggested structured followup was needed. A small number talked about their sense of loss at the completion of the sessions “*I miss it [support group] and feel as though I've been cut off a little bit.*” This was particularly pertinent for those who felt isolated or who had missed group sessions through illness and talked of unfinished business.

Only one participant reported that he did not enjoy the group meetings and found no support from them. He was comfortable with the technology and felt able to speak freely about his story, yet he felt unable to make any connections with the other participants and found no common stories to share or compare to help make sense of his accounts. One other participant was disappointed that he felt he couldn't discuss the use of alternative therapies “*I wanted to talk about alternative medicine … but I couldn't. It was sort of do not go there.*”

### 3.4. Supportive Care Strategies

Most of those involved reported that it was not until their support group meetings began that they were able to feel “normal” again. One woman explained that until she joined the support group, that she had lived with cancer for four years with issues left unanswered. Now, she felt that she could link with the experiences of others with advanced disease. Another said:

“*Just the feeling of being understood and having other people that had been through similar experiences, understand the impact that it had and the feelings that you have from that and the practicalities of it as well and being able to explore your feelings with them and also poke around about how bad it was.” *

All but four participants mentioned in interviews how good it was to be part of humorous debate. Two thought that their family and friends must think it inappropriate for them to laugh and joke because they were so ill. But several reported that it was during the banter in the support group meetings that they realized how much they missed being light-hearted: “*I love to listen to jokes and I love to laugh.*” Indeed, it seemed that being able to laugh, to be a part of a joke, and to laugh at the more difficult issues they faced was central to the support they felt from the groups. There were references in the interviews to group humor or “*in jokes.*” Mention of common group metaphors such “*lost at sea,*” “*missing the bus,”* “*winning the battle*” and a sense of common language among group participants through terms such as “*using the cancer tools*” and “*death-talk.*” Metaphors were adopted throughout the interviews as a way to report and describe feelings and experiences, especially those that related back to events within the support groups [15].

Examination of the latent content of interviews revealed two different motivations for involvement in these support groups. Two key narratives were identified: a dignity narrative and a deferral narrative. These explained participants' involvement in a support group, the type of support that was wanted, and appeared to shape their interactions with other group members.

The dignity narrative was typified by a focus on the future. Participants in whom this narrative was dominant wanted to be involved in a support group to help them as they worked out how to make meaning of their lives and attend to their self as someone living with cancer and facing death: “*Adjusting to the fact that this is terminal, makes you value each day, no matter what.*” This group of participants talked about their acceptance of their impending death in the interviews. One participant described their view of death as: “*Death is like passing through a door from time into eternity.*” Another related their focus on dying with dignity to conquering fear: “*I have no fear of death if it were to come soon.*"

Among these participants, the support group provided opportunities for “*hard to have*” discussions about end of life with others in a similar situation; this was particularly important when family and friends were uncomfortable discussing death and avoided “*death talk.*” For example, until joining his support group one person felt that he “*hardly existed*” in his family and friendship group as cancer and his impending death was absent from conversations with those close to him. Participation in a telephone support group gave him an opportunity to compare his experiences with others, helped him to understand the attitude of his family and friends, and gave relief to his feelings of isolation. Exploring dignity issues within a support group gave people courage to enter the risky territory of impeding death and to express things they were too scared to think about on their own. The dignity narrative was not necessarily only about acceptance but more about facing difficult realities: “*Good to face up to these important things because sooner or later we have to face it.*”

The dignity narrative also featured commitment to generativity and connections; in interviews, participants made references to different ways of leaving a legacy for loved ones, to sharing wisdom with other group members, and to affirming life's value. People talked about getting ideas from others on building memories and mementoes, particularly for their children and events where their absence will be missed. They discussed strategies and sought reassurance for preparing their children “*Just sit down with them and quietly tell them you may not always be there and in some way it helps to prepare them*” and got advice on things they could do to help others cope when they were gone, “*Go to the park where we always went and be with me, and remember me there.*”

People in a deferral narrative, in contrast, focused on coping with the present situation rather than looking to the future. Their attention was on living, not cancer and imminent death: “*It's so hard and exhausting to be thinking about cancer all the time. I am so much more than this disease. I will not let it take over my life. It can fit into my schedule.*” In the interviews, participants talked about how they were focused on keeping well and trying new therapies to manage multiple life demands:

“*I made a decision that I am not going to die from this cancer. Despite the poor prognosis of advanced breast cancer, I am simply not going to let it kill me. So my days are filled with positive thoughts and actions to get well and stay well.*”

A deferral narrative aligned with expectations that the support group would suggest ways to help cope with each day. It was clear from the interviews that the possibility of gaining support from others who shared common experiences was the main reason for joining a support group.

Recognition of the different narrative approaches allowed us to see how they complemented each other. One participant pointed this out clearly “*It's OK to face things head on or in bits and pieces. Strangely by voicing our differences we can sometimes realize we are, and need to be, different.*”

## 4. Discussion

This study confirmed findings from previous research that people with advanced cancer can find become involved in support groups to share experiences with others in similar situations [1–4]. This study adds new insight into different needs among those facing death by identifying two competing narratives, dignity and deferral, that show different needs and different expectations from support. The study also offers new understanding of the particular advantages of technological-based support for people facing untimely death.

### 4.1. Dignity and Deferral Narratives

The dignity narrative was consistent with findings of others who have explored in depth the various elements that make up a concern with a dignity in dying [17–19]. This narrative contained the three aspects identified by Tait et al., evaluation, transition, and legacy [20]. Participants focusing on dignity reported their illness as a means of transition to a dignified death and within this narrative, participation in a support group helped people to maintain a sense of continuity, to retain hope and self-regard; to come to an acceptance of their approaching death.

The deferral narrative represents the blurred boundaries between curative and palliative interventions experienced by people at the end-of-life and demonstrates the challenges for people facing untimely death. There is a well-cited literature on the death-denying culture of many western countries [21, 22], yet the interviews suggested that participants in this study were not denying their death but rather wanted to defer talking about it and focus on living a life in the present until death occurred [23]. They more typically focused on family and work responsibilities despite their advanced disease [24]. They looked to the support group for hope, help, and emotional support for the life they were living. These are recognised coping strategies among people facing death [16, 25, 26].

Analysis of the latent content in the transcripts demonstrated slippage between the two narratives as different issues were discussed. Participants from each narrative appeared to appreciate the presence of the other, as someone to both help and receive help from. Salander (1996) identified the development of the ability to hold apparently contradictory attitudes among people facing death as a reconstructive mechanism that facilitates coping; hope is retained despite an understanding of the reality of their situation [25].

### 4.2. Technology-Based Support: Meeting the Needs of Palliative Care Patients

The participants in this study were from support groups with four specific features: the groups were technology based; they were confined to people with advanced disease or in the palliative phase but were not specific to particular cancers; they were time limited; they were facilitated by a health professional.

We included both telephone and online groups in our definition of technology-based groups as these were the practical options available to potential participants in this study. Few studies have looked at telephone-based group support [27], possibly because widespread use of one-on-one telephone counseling in cancer services makes facilitated peer-based support seem as if it is a natural extension. The rapid uptake of online options for all types of support is also likely to explain the shift of research attention to this area. This study found that technology-based support groups offered particular benefits to people in the palliative stage of advanced cancer a group with a high level of unmet needs [28], which makes this an important finding from this study.

Consistent with other studies, study participants wanted to be with others with similar health experiences, including stage of disease progression[10] and age. It was notable that the average age of participants in this study (49 years) was more than 20 years younger than the average for cancer deaths in Australia [5]. The time-limited nature and lack of visibility experienced by participants in our study meant they did not witness the decline among fellow group members in late-stage groups [10]. Duration of support through such groups needs to be weighed with the time needed to develop rapport between group members [11].

Both types of technology-based support were especially useful when participants were undergoing treatment [29]. Technology removed geographical and social barriers to participation. Technology-based support groups saved time for participants in our study, which can be a precious resource at the end of life, and enabled better time management. Participants did not need to spend time travelling to get support, and the technologies made it feasible to connect with others with similar experiences no matter where they were, similar to reasons found in a small US study [24].

Consistent with other studies, a key benefit was anonymity [2], an advantage reported by participants from both types of groups. However, two key differences between the types of groups emerged as participants from online groups made two claims not reported by those from telephone groups. Online participants felt the technology allowed them to express emotions and also enabled them to revisit conversations by making use of the written threads that developed in the sessions.

Authors have argued that it is important for support groups to develop what some call “a group life” and others describe as “mutual aid” or “self help” [1, 12, 30]. We found evidence that a group life lingered through references to group humor and familiar metaphors that gave a sense of common language. These commonalities built a framework for group work and facilitated the support structure. Yet there was appreciation by some that their support group was a temporary group; it had worked well for them without disrupting their daily lives or existing relationships.

### 4.3. Study Limitations

This study was a small qualitative study at one time point. It would be useful to reproduce the study and include before-and-after interviews to ascertain if the narratives and experiences we identified are consistent patterns or were influenced by particular group interactions.

This can be a difficult group to study. This group is highly vulnerable and rightly subject to scrupulous ethical safeguards to protect privacy and ensure minimal impact from their participation in the study. This limited the information we could collect and report.

The research was undertaken in Melbourne, Australia where there are well-established cancer control programs and palliative care services funded by government. Melbourne is a multicultural city but its palliative care services are largely home-based, and, although responsive to different cultural expectations, people who use the Cancer Council services are more likely to come from the dominant white middle class. Different cultures and different sociodemographic groups may have different narratives that fit with other cultural patterns of managing death and dying. This study needs to be undertaken with groups from different cultures to ascertain the saliency of the narratives across cultures and sociodemographic groups.

### 4.4. Implications

As palliative care services reach more clients living longer with advanced disease, health professionals need strategies that assist them to provide social and emotional care to more people. Technology-based strategies provide opportunities to move beyond individualised care, and facilitate peer-to-peer support at the end of life, especially for younger clients or those socially or geographically isolated. These options are feasible for palliative care services to set up and acceptable to a group of clients.

## 5. Conclusion

As death from cancer becomes more common across the ageing western world, palliative care will need to take a more health promoting approach, extend into other aspects of health care and use innovative strategies to enhance social and emotional support among peers. In this study, we found particular benefits for palliative care clients through the use of technology-based support groups.

## References

[1] Ussher J, Kirsten L, Butow P, Sandoval M (2006). What do cancer support groups provide which other supportive relationships do not? the experience of peer support groups for people with cancer. *Social Science and Medicine*.

[2] Klemm P, Bunnell D, Cullen M, Soneji R, Gibbons P, Holecek A (2003). Online cancer support groups: a review of the research literature. *Computers, Informatics, Nursing*.

[3] Butow PN, Kirsten LT, Ussher JM, Wain GV, Sandoval M, Hobbs KM (2007). What is the ideal support group? Views of Australian people with cancer and their carers. *Psycho-Oncology*.

[4] Bell K, Lee J, Foran S, Kwong S, Christopherson J (2010). Is there an "ideal cancer" support group? key findings from a qualitative study of three groups. *Journal of Psychosocial Oncology*.

[5] Australian Institute of Health and Welfare and Australasian Association of Cancer Registries ( 2010). *Cancer in Australia: An Overview*.

[6] Ewers M, Schaeffer D (2007). Dying in Germany—consequences of societal changes for palliative care and the health care system. *Journal of Public Health*.

[7] Department of Health and Ageing ( 2011). *National Palliative Care Strategy 2010: Supporting Australians to Live Well at the End of Life*.

[8] Hsieh HF, Shannon SE (2005). Three approaches to qualitative content analysis. *Qualitative Health Research*.

[9] Till JE (2003). Evaluation of support groups for women with breast cancer: importance of the navigator role. *Health and Quality of Life Outcomes*.

[10] Vilhauer RP (2011). ‘Them’ and ‘us’: the experiences of women with metastatic disease in mixed-stage versus stage-specific breast cancer support groups. *Psychology and Health*.

[11] Gottlied BH, Wachala ED (2007). Cancer support groups: a critical review of empirical studies. *Psycho-Oncology*.

[12] Emslie C, Whyte F, Campbell A (2007). ’I wouldn’t have been interested in just sitting round a table talking about cancer’; exploring the experiences of women with breast cancer in a group exercise trial. *Health Education Research*.

[13] Graneheim UH, Lundman B (2004). Qualitative content analysis in nursing research: concepts, procedures and measures to achieve trustworthiness. *Nurse Education Today*.

[14] Cohen D, Crabtree B (2008). Evaluative criteria for qualitative research in health care: controversies and recommendations. *Annals of Family Medicine*.

[15] Leydon GM, Boulton M, Moynihan C (2000). Cancer patients’ information needs and information seeking behaviour: in depth interview study. *British Medical Journal*.

[16] Sand L, Olsson M, Strang P (2009). Coping strategies in the presence of one’s own impending death from cancer. *Journal of Pain and Symptom Management*.

[17] Patton M ( 2002). *Qualitative Research and Evaluation Methods*.

[18] Nguyen HQ, Carrieri-Kohlman V, Rankin SH, Slaughter R, Stulbarg MS (2004). Internet-based patient education and support interventions: a review of evaluation studies and directions for future research. *Computers in Biology and Medicine*.

[19] Ussher J, Kirsten L, Butow P, Sandoval M (2006). What do cancer support groups provide which other supportive relationships do not? the experience of peer support groups for people with cancer. *Social Science and Medicine*.

[20] Tait GR, Schryer C, McDougall A, Lingard L (2011). Exploring the therapeutic power of narrative at the end of life: a qualitative analysis of narratives emerging in dignity therapy. *BMJ Supportive and Palliative Care*.

[21] Hack TF, Carlson L, Butler L (2011). Facilitating the implementation of empirically valid interventions in psychosocial oncology and supportive care. *Supportive Care in Cancer*.

[22] Carlander I, Ternestedt BM, Sahlberg-Blom E, Hellström I, Sandberg J (2011). Being me and being us in a family living close to death at home. *Qualitative Health Research*.

[23] Reb AM (2007). Transforming the death sentence: elements of hope in women with advanced ovarian cancer. *Oncology Nursing Forum*.

[24] Im EO, Chee W, Lim HJ, Liu Y, Guevara E, Kyung SK (2007). Patients’ attitudes toward internet cancer support groups. *Oncology Nursing Forum*.

[25] Salander P, Bergenheim T, Henriksson R (1996). The creation of protection and hope in patients with malignant brain tumours. *Social Science and Medicine*.

[26] Rustøen T, Cooper BA, Miaskowski C (2010). The importance of hope as a mediator of psychological distress and life satisfaction in a community sample of cancer patients. *Cancer Nursing*.

[27] Hoey LM, Ieropoli SC, White VM, Jefford M (2008). Systematic review of peer-support programs for people with cancer. *Patient Education and Counseling*.

[28] Rainbird K, Perkins J, Sanson-Fisher R, Rolfe I, Anseline P (2009). The needs of patients with advanced, incurable cancer. *British Journal of Cancer*.

[29] Van Uden-Kraan CF, Drossaert CHC, Taal E, Shaw BR, Seydel ER, Van de Laar MAFJ (2008). Empowering processes and outcomes of participation in online support groups for patients with breast cancer, arthritis, or fibromyalgia. *Qualitative Health Research*.

[30] Barak A, Boniel-Nissim M, Suler J (2008). Fostering empowerment in online support groups. *Computers in Human Behavior*.

